# *Acinetobacter* sp. DW-1 immobilized on polyhedron hollow polypropylene balls and analysis of transcriptome and proteome of the bacterium during phenol biodegradation process

**DOI:** 10.1038/s41598-017-04187-6

**Published:** 2017-07-07

**Authors:** Qihui Gu, Qingping Wu, Jumei Zhang, Weipeng Guo, Huiqing Wu, Ming Sun

**Affiliations:** 1 School of Bioscience and Bioengineering, South China University of Technology, Guangzhou, 510006 P.R. China; 2 Guangdong Institute of Microbiology, State Key Laboratory of Applied Microbiology Southern China; Guangdong Provincial Key Laboratory of Microbial Culture Collection and Application, Guangdong Open Laboratory of Applied Microbiology, 510070 Guangzhou, P.R. China; 3 Guangzhou Institute of Chemistry, Chinese Academy of Sciences, Guangzhou, 510650 P.R. China

## Abstract

Phenol is a hazardous chemical known to be widely distributed in aquatic environments. Biodegradation is an attractive option for removal of phenol from water sources. *Acinetobacter* sp. DW-1 isolated from drinking water biofilters can use phenol as a sole carbon and energy source. In this study, we found that Immobilized *Acinetobacter* sp. DW-1cells were effective in biodegradation of phenol. In addition, we performed proteome and transcriptome analysis of *Acinetobacter* sp. DW-1 during phenol biodegradation. The results showed that *Acinetobacter* sp. DW-1 degrades phenol mainly by the ortho pathway because of the induction of phenol hydroxylase, catechol-1,2-dioxygenase. Furthermore, some novel candidate proteins (OsmC-like family protein, MetA-pathway of phenol degradation family protein, fimbrial protein and coenzyme F390 synthetase) and transcriptional regulators (GntR/LuxR/CRP/FNR/TetR/Fis family transcriptional regulator) were successfully identified to be potentially involved in phenol biodegradation. In particular, MetA-pathway of phenol degradation family protein and fimbrial protein showed a strong positive correlation with phenol biodegradation, and Fis family transcriptional regulator is likely to exert its effect as activators of gene expression. This study provides valuable clues for identifying global proteins and genes involved in phenol biodegradation and provides a fundamental platform for further studies to reveal the phenol degradation mechanism of *Acinetobacter* sp.

## Introduction

Phenol is a toxic compound that is widely distributed in nature, especially in aquatic environments. Phenolic compounds are used as raw materials for the production of various products in different industries, including oil refinement, pharmaceuticals, pesticides, resin products, steel mills, coke oven plants, and leather production^1, 2^. However, because of overuse and uncontrolled emissions, phenol has drawn increasing attention as a threat to human health and is on the priority list of highly hazardous chemicals in China. In the National Chinese Standards for Drinking Water Quality (GB5749-2006), the acceptable concentration of volatile phenol is limited to 2 ppb. Therefore, it is necessary to remove phenol from the aquatic environment. Biodegradation technology is a potentially attractive tool for removal of environmental pollutants because it has low cost, saves energy, is efficient, and does not lead to secondary pollution^3^.

*Acinetobacter* sp. DW-1(GenBank accession number KU499947), which was isolated from a biological active carbon (BAC) filter in a drinking water plant in our laboratory^4^, is able to grow on mineral salt medium (MSM) with phenol as a sole carbon and energy source. *Acinetobacter* spp. are commonly found in soil and aquatic environments, where they comprise a significant part of the total heterotrophic, aerobic population. Moreover, they are able to grow in low nutrient media containing inorganic sources of nitrogen and a variety of simple compounds as carbon and energy source^5^. *Acinetobacter* spp. have been attracting increasing attention in both environmental and biotechnological applications^6^. Previous studies demonstrated that immobilized cells often exhibited better performance in biodegradation than free-bacteria^7, 8^. Moreover, immobilized bacteria could strengthen the resistance to environmental stress compared with free bacteria^9^. Therefore, it is of great importance to investigate the phenol biodegradation capacity of immobilized *Acinetobacter* sp. DW-1. Meanwhile, antimicrobial susceptibility data for *Acinetobacter* spp. isolated from drinking water environment is still scarce. Therefore the goal of the current antimicrobial susceptibility experiments was to determine the controllability of this strain when it is applied in water treatment.

To date, the aerobic catabolic pathway for phenol has been extensively investigated in the *Pseudomonas* ^10–12^ and *Acinetobacter* ^13, 14^ genera which often isolated from polluted or contaminated environmental samples. Therefore, they had a strong innate tolerance to pollutants from different sources, and can metabolize these pollutants. However, how the indigenous bacterium which isolated from low-nutrient and micro-pollution environment responses to phenol, and the reasons for variations in the level of expression of important enzymes and regulatory factors during catabolism are still unclear. More work is necessary to thoroughly characterize the catabolic pathways of phenol and global metabolism to elucidate phenol degradation mechanism. To the best of our knowledge, proteome and transcriptome approaches were powerful to discover molecular markers and elucidation of functional mechanisms^15, 16^. However, using alone approach could not obtain sufficient information. Different types of data often provide complementary information to the elucidation of mechanisms, so that the detection bias from each of the technologies can be avoided^17^. They can provide detailed and comprehensive information on the biodegradation process^18, 19^. However, currently the information on phenol biodegradation by *Acinetobacter* sp. was mainly evaluated by proteome^20–22^, there is no reports about using transcriptome approach to investigate phenol biodegradation by *Acinetobacter* sp. Therefore, it is of interest to determine the catabolic pathway and to elucidate the mechanism of phenol biodegradation by *Acinetobacter* sp. DW-1 using both proteomic and transcriptomic analyses. Proteomic techniques are effective methods for the detection of detailed differences in protein expression under alternative growth condition^23, 24^. Among proteomic techniques, two-dimensional electrophoresis (2-DE) is widely used to analyze bacterial proteins; for example, the log to stationary growth phase in *Lactobacillus plantarum* cells^25^, differentiation of low-Mr-secreted protein species in mycobacteria^26^, and response to alkaline stress induced by root canal bacteria in biofilms^27^ have been analyzed by proteomic methods. Moreover, differential gene expression in response to phenol and catechol has revealed various metabolic activities for the degradation of aromatic compounds in *Bacillus subtilis* ^28^. Similarly, transcriptome sequencing is a good tool to comprehensively study the differences in genes expressed under different treatments^29–32^.

The aim of this study was to better understand the biofilm-forming ability of *Acinetobacter* sp. DW-1 on carrier materials, the controllability of this strain, and then to find novel candidate proteins or related genes so as to further reveal phenol degradation mechanism, ultimately to evaluate the potential for its practical use in bioremediation applications. These aims were achieved using drug sensitivity test and by comparing the proteome and transcriptome of strains grown on different carbon sources and analyzing the expression of major metabolic enzymes during the phenol degradation process. To the best of our knowledge, this is the first attempt to systematically investigate the biofilm-forming ability of the native bacterium isolated from drinking water biofilter on carrier materials, and to assess the controllability of indigenous bacterium before practical application. This study provides abundant both proteome and transcriptome data for phenol biodegradation by *Acinetobacter* sp. The candidate genes involved in the biosynthesis and transcriptional regulation of *Acinetobacter* sp. were obtained in this study. This provides information that increases our understanding of the mechanism of phenol regulation by *Acinetobacter* sp. The global gene regulation and interplay during phenol degradation process remain largely unaddressed. With the present study we provide novel information about the gene regulation relevant to such a biodegradation process by *Acinetobacter* sp. The comparative analysis of the proteome and transcriptome data of phenol biodegradation by *Acinetobacter* sp. DW-1, lays the foundation for elucidating the mechanism of phenol degradation, and thus, enables future improvements in phenol biodegradation.

## Materials and Methods

### Immobilization of *Acinetobacter* sp. DW-1 cells

Prepared sterilized polyhedron hollow polypropylene balls (Jingying, Yixing, China) were placed into a 500 mL flask containing 200 mL of MSM medium supplemented with 4 mM phenol as a sole carbon and energy source. This mixture was incubated without motion at 30 °C to allow *Acinetobacter* sp. DW-1 isolated in our laboratory cells to attach to the polyhedron hollow polypropylene balls. The balls were withdrawn at 24, 48, and 72 h using sterile forceps. The balls were then rinsed with sterile MSM to remove any *Acinetobacter* sp. DW-1 cells not sufficiently adhered to their surfaces. Next, the rinsed balls were examined by scanning electron microscopy (SEM).

### Detection of biofilm by scanning electron microscopy and confocal laser scanning microscopy

*Acinetobacter* sp. DW-1 cells immobilized on polyhedron hollow polypropylene balls were examined using a S-3000N scanning electron microscope (Hitachi, Tokyo, Japan). In preparations, cells were fixed in 0.1 M phosphate buffer solution (PBS) containing 3% (v/v) glutaraldehyde at 4 °C for 5 h. The samples were then washed with PBS (pH 7.0) six times (20 min each). The samples were then gradually dehydrated with ethanol. Dehydrated cells were filtered through a 0.2 µm polycarbonate filter, dried with a CO_2_-critical point dryer, coated with gold and subsequently observed by SEM at 20 kV. Confocal laser scanning microscopy (CLSM) (Zeiss, Berlin, Germany) analysis was performed using a LIVE/DEAD® BacLight™ Bacterial Viability and Counting Kit (Invitrogen, Carlsbad, USA) with an appropriate mixture of the SYTO 9 and propidium iodide (PI).

### Adennosine tri-phosphate (ATP) analysis

ATP measurements to estimate the active biomass of *Acinetobacter* sp. DW-1 immobilized on polyhedron hollow polypropylene balls were obtained. For the preparations, the balls were withdrawn from the incubated flask, and then rinsed with PBS three times to remove the unattached cells. Subsequently, the rinsed polyhedron hollow polypropylene balls with PBS into an ultrasonic cleaner for 5 min. The supernatant was used for ATP measurement which was developed by our laboratory as described elsewhere in detail^33^.

### Investigation of phenol biodegradation by immobilized *Acinetobacter* sp. DW-1 cells

Six glass columns (inner diameter = 40 cm) with a working volume of 450 mL each were filled with sterile polyhedron hollow polypropylene balls. Three columns were used as controls, whereas the other three were used to immobilize *Acinetobacter* sp. DW-1. Sand-filtered water samples with initial phenol concentrations of 4, 2, and 1 mM were applied to the columns using a circulating water pump (Sensen, Zhoushan, China). The empty bed contact time was 18 min. The entire water treatment process was maintained at room temperature. Effluent water from samples with different initial phenol concentrations was collected for phenol detection after 1 h. The phenol concentration was determined as described elsewhere^4^.

### Transmission electron microscopy

*Acinetobacter* sp. DW-1 cultures were grown in MSM supplemented with 4 mM phenol as a single carbon and energy source at 30 °C for 30 h; then, several drops of cell suspension were applied to a collodion-coated copper grid of mesh size 200 for 5 min. After a brief wash with water, the grid was immersed in filtered 3% phosphotungstic acid (pH 7.0) for 2 min. The grid was then removed, the excess stain was wicked away with filter paper, and the grid was washed with water and dried at room temperature for 45 min. Specimens were observed under an H7500 transmission electron microscope (TEM; Hitachi) operating at 80 kV.

### Antimicrobial susceptibility test

Antibiotic susceptibility tests were performed using the agar disk diffusion method^34^, following the guidelines of the Clinical and Laboratory Standards Institute. Nineteen antibiotic-containing disks (Oxoid, Basingstoke, UK) were performed for 17 antibiotics that listed in Supplementary Information. Antimicrobial susceptibility tests were performed as previously described^35^.

### Bacterial culture conditions and phenol biodegradation

Proteins prepared from *Acinetobacter* sp. DW-1 cells were inoculated into R2A fluid nutrient medium^36^ and incubated until the culture reached exponential phase; then, 200 μL of R2A-preculture was inoculated into an Erlenmayer flask (1000 mL) containing MSM supplemented with acetate (4 mM) as the sole carbon source. The composition of MSM was as follows (mg L^−1^): MgSO_4_.7H_2_O, 0.1; NaCl, 0.2; NH_4_Cl, 0.5; Na_2_HPO_4_.12H_2_O, 0.5; KH_2_PO_4_, 0.5; FeCl_3_.6H_2_O, 0.1; and CaSO_4_.H_2_O, 0.1. Cultures were incubated at 30 °C and shaken at 120 rpm in an environmental chamber until they reached standardized optical densities at of 0.7–0.8 600 nm (OD_600_). Subsequently, phenol was added to the cultures to obtain a final concentration of 4 mM. Unsupplemented cultures served as controls. Cells from the control and phenol- supplemented cultures were harvested when the phenol biodegradation rate reached a maximum. However, to determine growth curves, we performed viable cell counts during the phenol biodegradation process by withdrawing 1 mL samples of culture every 2 h under sterile conditions, diluting them up to 10^6^ in Eppendorf tubes, and plating the dilutions in triplicate on Plate Count Agar using a glass L-shaped rod. After 48 h of incubation, colonies were counted to identify dilutions producing colony numbers between 40 and 400 (to facilitate interpretation of results). Phenol degradation was evaluated by collecting samples of MSM solution at every two hours for acetate as well as phenol quantification. The determination of acetate and phenol concentration were performed as described previously^37, 38^. Meanwhile, the major intermediate products of phenol which including catechol and *cis,cis*-muconate were also investigated during the biodegradation process, and the detection methods of them were according to previous studies^39, 40^.

### Preparation of crude protein extracts

Cells were harvested by centrifugation at 12,000 g for 10 min at 4 °C, washed twice in MSM, and sonicated with an ultrasonic processor (Sonics VCX130PB, USA) for a total of 20 min at 20 kHz with intervals of 1 min, where the cells were kept on ice. The sonicated solution was then clarified by centrifugation at 12,000 g for 1 h at 4 °C. The protein concentration was determined using a Non-Interfering Protein Concentration Determination Kit (Sangon, Shanghai, China).

### Conditions for 2-DE

Protein samples (800 µg) were mixed with rehydration solution (0.2% (v/v) Bio-Lyte ampholyte (pH 3–10), 8 M urea, 4% (w/v) CHAPS, 1% (w/v) DTT, and 0.01% (w/v) bromophenol blue) in total volume of 350 µL. Immobiline TM Dry Strip IPG strips (17 cm, pH 4–7) were passively hydrated at 16 °C for 13 h. IEF and two-dimensional SDS-polyacrylamide gel electrophoresis (PAGE) were performed as described elsewhere in detail^41^.

### Image analysis and statistics

Gels were stained with Coomassie brilliant blue G-250, and triplicate gels were scanned using a densitometric Image Scanner (GE Healthcare). The raw images were analyzed using Image Master TM platinum version 7.0 (GE Healthcare). For each comparison of cells grown on acetate with those grown on acetate plus phenol), the 2-D gels were analyzed in triplicate. The method to screen for phenol-induced proteins was consulted previous literature^42^.

### Identification of proteins by MS

To identify differentially expressed proteins, spots of interest were excised manually from Coomassie brilliant blue stained polyacrylamide gels and subjected to in-gel digestion as previously described^43^. The excised proteins were analyzed using a 4700 MALDI-TOF/TOF proteomics analyzer (Applied Biosystems). A peptide mass fingerprinting search and a combined search (+MS/MS) were performed using GPS Explorer™ software (Applied Biosystems) in non-redundant NCBI database of proteins using MASCOT searching engine as described previously^44^.

### RNA extraction and sequencing

*Acinetobacter* sp. strain DW-1 cells in exponential phase growing on acetate or acetate plus phenol were collected and used for total RNA extraction. RNA of each sample was extracted using a MiniBEST Universal RNA Extraction Kit (TaKaRa, Dalian, China) according to the manufacturer’ instructions. The assessing of RNA concentration and quality and RNA sequencing as described previously^45^.

### Transcriptome assembly and analysis

Raw sequences were first quality-filtered, and the differential gene expression analysis was then performed. The differentially expressed genes were subjected to GO (http://www.geneontology.org/) and KEEG (http://www.genome.jp/kegg/) analyses. Differentially expressed genes (DEGs) were obtained based on the Reads Per Kilobase of exon model per Million (RPKM), followed by a multiple hypothesis testing, false discovery rate (FDR) control to correct the p-value. Genes with an FDR value < 0.05 and |log2FC| > 1 were classified as differentially expressed.

### Validation of RT-qPCR

After RNA extraction, complementary DNA(cDNA) was synthesized using total RNA with a PrimeScript RT reagent kit (TaKaRa, Dalian, China), and then the cDNA product was amplified by RT-PCR. 16sRNA was used as an internal reference in all reactions. Real-time quantification of mRNA was performed on an Eppendorf Realplex (4) PCR system (Eppendorf, Hamburg, Germany) using SYBR Premix ExTaq II kits (TaKaRa, Dalian, China). The comparative Ct method was employed for quantification of target gene expression that was normalized to 16sRNA expression and relative to the calibrator. Data were expressed as the fold change (T/CK) = 2−^ΔΔCt^. A list of all primers used in this study is presented in Supplementary Table S6.

## Results and Discussion

### Detection of *Acinetobacter* sp. DW-1 biofilm formation and phenol degradation test

**Figure 1:**
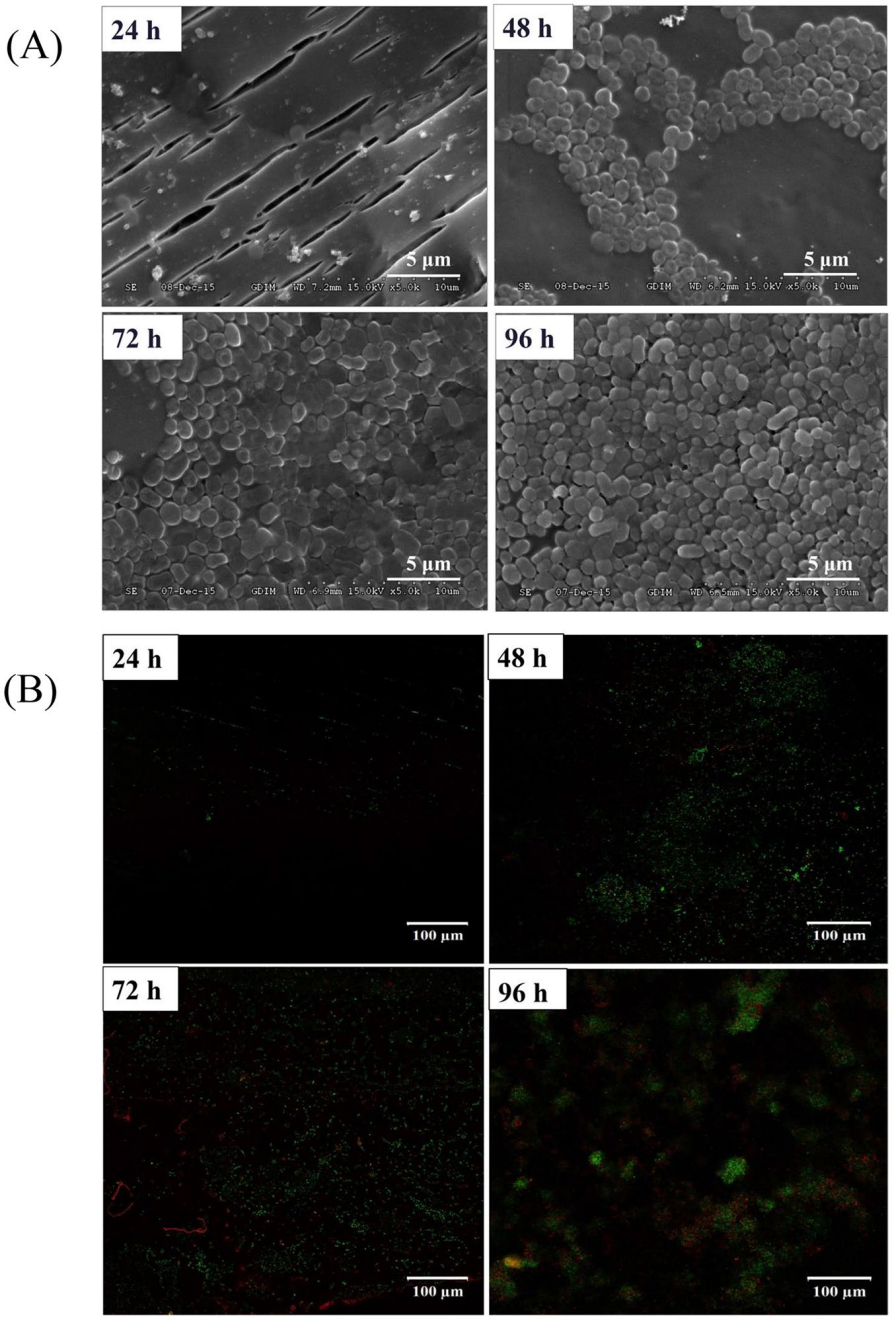
Growth of an *Acinetobacter* sp. DW-1 biofilm on polyhedron hollow polypropylene balls visualized by scanning electron microscopy (**A**) and confocal laser scanning microscopy (**B**).

**Figure 2:**
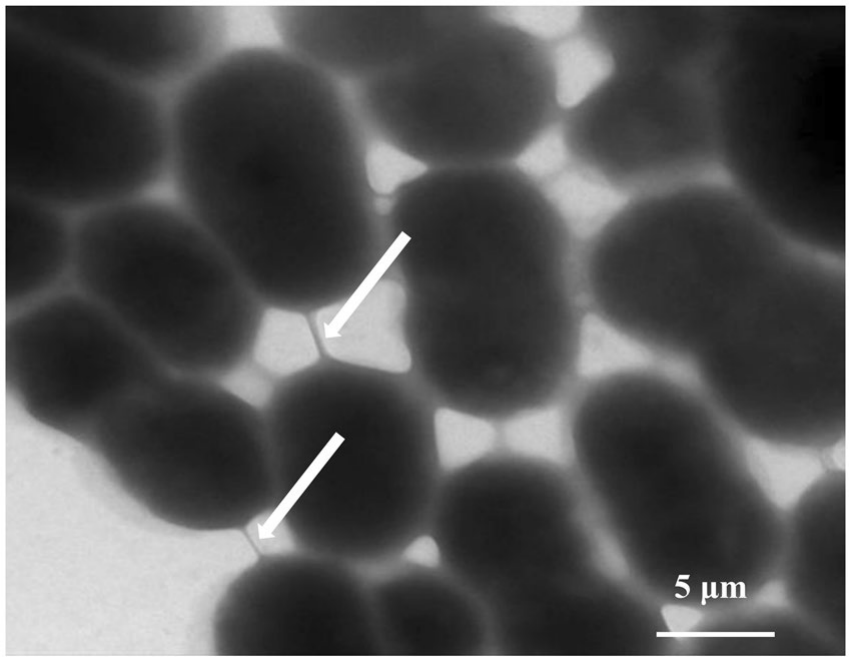
TEM image of *Acinetobacter* sp. DW-1 grown in MSM supplemented with 4 mM phenol as single carbon and energy source. The arrows indicate anchor-like appendages among cells.

According to previous experiment (Supplementary methods 1.2), we found that immobilized cells of *Acinetobacter* sp. DW-1 showed better activity for the phenol biodegradation than free cells (Supplementary Fig. S1). Therefore, we chose immobilized cells to study in this study. SEM visualization demonstrated that *Acinetobacter* sp. DW-1 successfully attached to polyhedron hollow polypropylene balls and grew well (Fig. 1A). The surface of the polyhedron hollow polypropylene balls was fully covered with *Acinetobacter* sp. DW-1cells after 96 h immobilization. Confocal laser scanning microscopy confirmed that the quantity of *Acinetobacter* sp. DW-1 cells on the polypropylene balls increased over time; however, the number of dead cells (red spots) also increased gradually (Fig. 1B). The results were in agreement with those obtained by measuring the ATP concentration, which demonstrated that the highest biological activity was obtained for *Acinetobacter* sp. DW-1 cultivated for 72 h, and that the biological activity decreased when the cultures were grown for 96 h (Table 1). Figure 1B shows that the quantity of *Acinetobacter* sp. DW-1 cells increased as the cultivation increased, but the number of dead cells (red spots) also increased gradually. The results agree with the ATP results (Table 1), where *Acinetobacter* sp. DW-1 cultivated for 72 h showed the highest biological activity. However, the biological activity decreased when the cultures were grown to 96 h (Table 1). TEM images of *Acinetobacter* sp. DW-1 (Fig. 2) revealed anchor-like appendages connecting cells, which may contribute to the capability of *Acinetobacter* sp. DW-1 to adhere to solid surfaces^46^. These results indicated that the *Acinetobacter* sp. DW-1 biofilm reached an optimal state after 72 h of cultivation. Therefore, we selected this stage for assessment of phenol biodegradation by the attached biomass.

**Table 1:** Adenosine tri-phosphate (ATP) analysis of *Acinetobacter* sp. DW-1 immobilized on polyhedron hollow polypropylene balls at different times.

Time (h)	24	48	72	96
ATP (mol/g)	8.41 × 10^−8^ ± 1.23	3.36 × 10^−7^ ± 4.16	2.11 × 10^−6^ ± 5.02	6.28 × 10^−8^ ± 3.46

**Figure 3:**
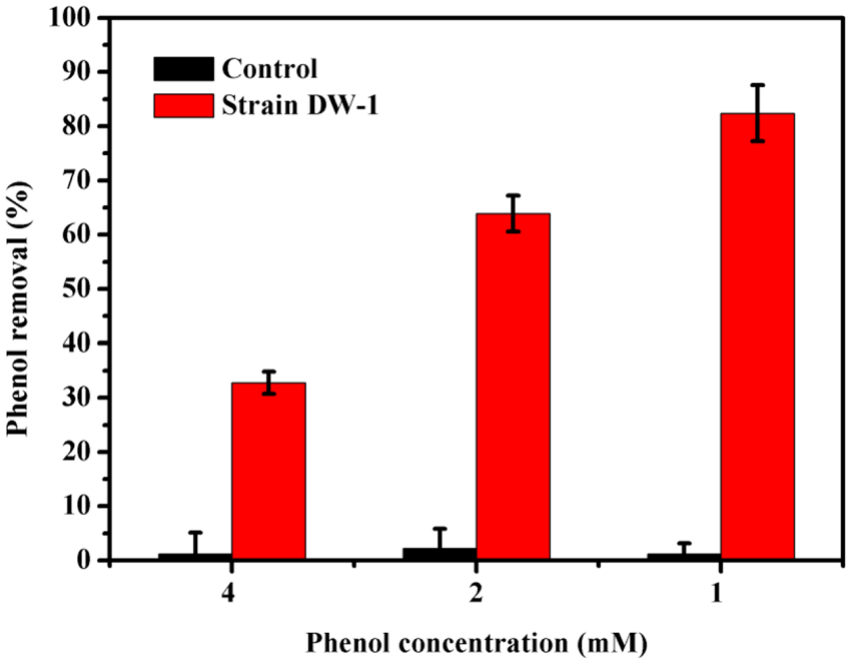
Phenol degradation by *Acinetobacter* sp. DW-1 immobilized on polyhedron hollow polypropylene balls.

*Acinetobacter* sp. DW-1 immobilized on polyhedron hollow polypropylene balls exhibited good performance in phenol biodegradation (Fig. 3). At a phenol concentration of 1 mM, *Acinetobacter* sp. DW-1 degraded approximately 80% of phenol, and at 4 mM, approximately 33%. The results from the control samples indicated that the polyhedron hollow polypropylene balls adsorbed negligible amounts of phenol, demonstrating their suitability as a material for investigation of degradation by *Acinetobacter* sp. DW-1. Therefore, our results indicate that *Acinetobacter* sp. DW-1 has a strong ability to form biofilms and degrade phenol.

### Antimicrobial susceptibility test

*Acinetobacter* sp. DW-1 was susceptible to the majority of antimicrobials included in the test panel. However, it was resistant to chloramphenicol and aztreonam (Table 2). The resistance profile of *Acinetobacter* sp. DW-1, which was isolated from an activated carbon filter in a drinking water plant, was different from that of clinically isolated *Acinetobacter* strains. Previous reports indicate that imipenem is highly active against all *Acinetobacter* isolates obtained from patients^47–49^. However, in the present study, *Acinetobacter* sp. DW-1 was susceptible to imipenem, but resistant to chloramphenicol. These results were consistent with those obtained using *Acinetobacter* sp. isolated from wastewater^50^, mineral water^51^ and drinking water^52^, all of which showed resistance to chloramphenicol. In general, strain DW-1 was susceptible to antibiotics and, therefore, controllable. Moreover, as an indigenous bacterium from an aquatic environment, strain DW-1 was able to adapt to oligotrophic conditions. In summary, strain DW-1 shows strong potential for application in the removal of phenol from water.

**Table 2:** Results of antibiotic susceptibility tests of *Acinetobacter* sp. DW-1.

Antimicrobial agents	Breakpoints (mm)		Tolerance (Breakpoints (mm))	
	Susceptible (S)	Intermediate (I)	Resistant (R)	
Kanamycin(K)	≥18	14–17	≤13	S(26.96)
Streptomycin(S)	≥15	11–14	≤10	S(16.09)
Tetracycline(TE)	≥19	15–18	≤14	S(20.02)
Ampicillin(AMP)	≥17	14–16	≤13	S(14.72)
Chloramphenicol(C)	≥26	20–25	≤19	R(15.84)
Ciprofloxacin(CIP)	≥21	16–20	≤15	S(25.69)
Meropenem(MEM)	≥16	14–15	≤13	S(29.55)
Cephalothin(KF)	≥18	15–17	≤14	S(21.52)
Tobramycin(TOB)	≥15	13–14	≤12	S(21.61)
Gentamicin(CN)	≥15	13–14	≤12	S(20.21)
Ceftazidime(CAZ)	≥21	18–20	≤17	I(18.86)
Cefotaxime(CTX)	≥23	15–22	≤14	I(17.67)
Imipenem(IPM)	≥16	14–15	≤13	S(28.67)
Cefepime(FEP)	≥18	15–17	≤14	S(19.45)
Trimethoprim-Sulfamethoxazoie(SXT)	≥16	11–15	≤10	S(19.07)
Levofloxacin(LEV)	≥17	14–16	≤13	S(29.38)
Aztreonam (ATM)	≥21	18–20	≤17	R(0)

### Bacterial strains and culture conditions

**Figure 4:**
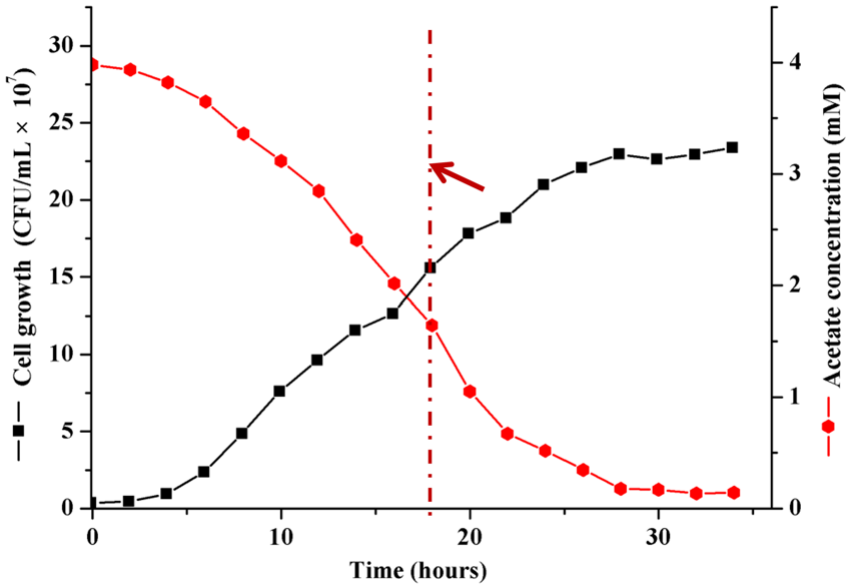
Growth curves of *Acinetobacter sp*. DW-1. Cells were grown at 30 °C in MSM with 4 mM acetate as the sole carbon and energy source. Growth was followed using the plate counting method. Cells were harvested from cultures for subsequent experiments after 18 h incubation (red arrow).

**Figure 5:**
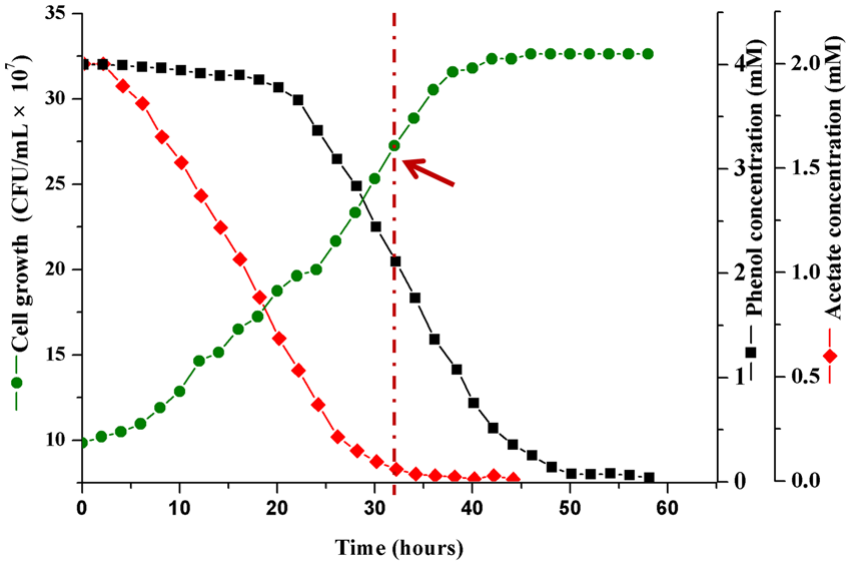
Growth curves of *Acinetobacter sp*. DW-1. Cells were grown at 30 °C in MSM with 4 mM acetate and 4 mM phenol. Growth was assessed using the plate counting method. Cells were harvested from cultures that after 32 h incubation (red arrow) following phenol supplementation for subsequent experiments.

To evaluate phenol-induced alterations occurring in the proteome, of *Acinetobacter* sp. DW-1, samples were collected during the exponential growth phase from cultures grown on acetate, or acetate plus phenol, for proteomic analysis. These conditions were selected with the aim of obtaining relevant information about the global response of proteins in cells adapted to consumption of phenol. *Acinetobacter* sp. DW-1 required 30 h to degrade 4 mM acetate in its growth medium (Fig. 4). However, when the cultures were provided with a mixture of acetate and phenol, the degradation rate of acetate was reduced compared to growth on acetate as the single carbon source (Fig. 5). Therefore, high concentrations of phenol inhibit the growth of cells, as previously reported^53^. By contrast, phenol consumption rates remained low until acetate levels in the growth media were depleted, after which the rate of phenol degradation increased rapidly (Fig. 5). These results are consistent with previous reports demonstrating a hierarchy of substance preference: benzoate > acetate > phenol^54^. In addition, the major intermediate products of phenol including catechol and *cis,cis*-muconate were observed in medium simultaneously (Supplementary Figure 2). However, the concentrations of intermediates were always less than 10 mg/L under the conditions studied, and they quickly disappeared when phenol completely degraded. This data is agreement with previous report^55^.

### Identification of proteins by mass spectrometry

**Figure 6:**
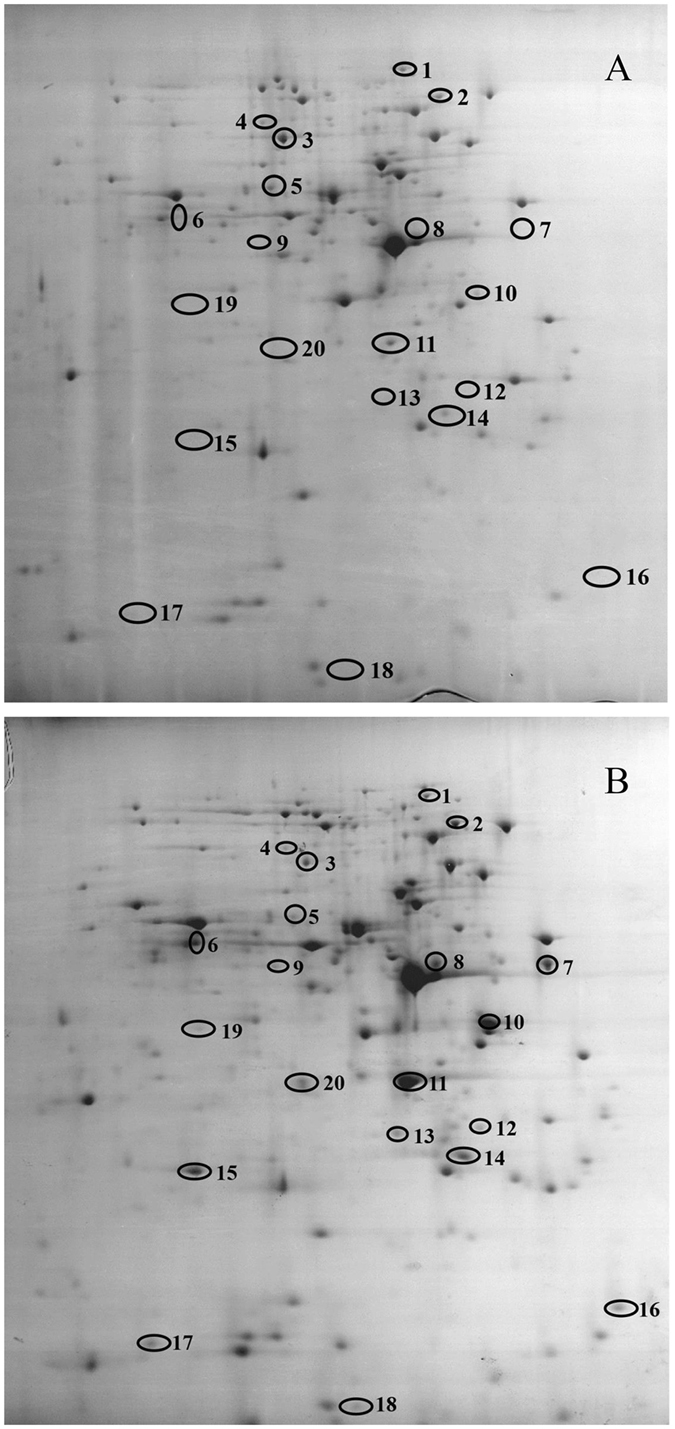
2-D PAGE gels of soluble proteins from *Acinetobacter* sp. DW-1 cells grown in MSM supplemented with 4 mM acetate (**A**) or 4 mM acetate and 4 mM phenol (**B**). pH 4–7 (from right to left).

To determine which proteins were induced by culture of *Acinetobacter* sp. DW-1 with phenol, comparative 2-DE was performed in the pH range of 4–7 using crude extract from cells grown on acetate as controls (Fig. 6A). Because phenol is metabolized to acetyl CoA, which then enters the tricarboxylic acid cycle, acetate was a useful carbon source for comparison with the known proteomic effects of phenol. Additionally, phenol is always decomposed into acetate, succinate, pyruvic acid, and acetaldehyde via the ortho- and meta-pathways during phenol biodegradation.

As shown in Fig. 6, bacterium grown on acetate plus phenol expressed more proteins than bacterium grown on acetate. Over 500 proteins were separated on the pH 4–7 acetate 2-D gels, and over 600 proteins were separated on the acetate plus phenol 2-D gels. In total 20 protein spots with significant differences between acetate and acetate plus phenol 2-D gels were identified by MALDI-TOF/TOF MS (Table 3), with 15 up-regulated protein spots, and 11 spots that were only present in samples grown on acetate plus phenol. This difference was partly attributed to the ability of acetate to easily enter most central metabolic cycles because of its C2 molecule. To grow on a phenol substrate, *Acinetobacter* sp. DW-1 must activate additional enzymes (Table 3). Among the proteins identified in cells growing on phenol, six protein spots corresponded to enzymes involved in the phenol catabolic pathway. Most of these enzymes were found only in samples grown on phenol-containing media, including phenol hydroxylase, 3-oxoadipate CoA-transferase subunit B, 3-oxoadipate CoA-transferase subunit A, and the muconate and chloromuconate cycloisomerases family protein, 3-oxoadipyl-CoA thiolase. Among these enzymes, previous studies indicate that phenol hydroxylases are the key rate-limiting enzymes catalyzing the initial reaction in phenol degradation, and can be classified into three types: single-, double-, and multi-component^56, 57^. Multi-component phenol hydroxylases are widely distributed in gram-negative bacteria, such as *Pseudomonas* sp. OX1 and *Acinetobacter radioresistens* ^58, 59^. Two-component phenol hydroxylases are exclusively distributed in gram-positive bacteria, such as *Bacillus thermoglucosidasius* A7^60^. A single-chain phenol hydroxylases isolated from the eukaryote *Trichosporon cutaneum* has been reported^61^. Catechol-1, 2-dioxygenase (C12O) is another important enzyme in the phenol degradation pathway, as the second enzyme of phenol metabolism, and has been used as a marker for this pathway^62^. Catechol is cleaved by C12O, and the cleavage product, cis, cis-muconate, is transformed via 1-ketoadipate-enol-lactone to succinate and acetyl CoA^63^. In the present study, the expression of C12O in samples grown on phenol-containing medium was 24.5042-fold higher than that in cells grown on acetate alone. C12O has been discovered in many bacteria grown on aromatic compounds, including phenol, as the sole carbon source. Interestingly, muconate and chloromuconate cycloisomerase family protein was also upregulated when bacteria grew on acetate plus phenol. Muconate cycloisomerase (EC 5.5.1.-) is the third enzyme of phenol metabolism^62^, and has been purified from extracts of *Rhodococcus erythropolis* 1CP cells grown on benzoate. Furthermore, 3-oxoadipate CoA-transferase subunit B (Fragment) has been described among benzoate-induced proteins of *Pseudomonas. putida* KT2440^12^, and among aromatic and choloroaromatic-induced proteins of *Pseudomonas* sp. Strain B13, which are necessary to reach the Krebs cycle after the convergence of pathways used for the degradation of aromatic compounds^64^. The function of 3-oxoadipate CoA-transferase, an important enzyme in benzoate catabolism, is to catalyze the conversion of 3-oxadipate to 3-oxoadipyl-CoA, in the catechol branch of the beta-ketoadipate pathwaty. Moreover, it is necessary in the chloroaromatic degradation pathway^65^.

**Table 3:** Identification and characterization of differential expression proteins by MALDI/TOF/TOF MS between acetate or phenol as the sole carbon.

Identified proteins	Mas score^a^	Spot No.^b^	GI No.	Calculated pI value ^c^	Sequence coverage (%)	Mass (Da)^d^	Proteins location	Express ion of acetate/acetate plus phenol (fold change)
Aconitate hydratase B	169	1	1310689	4.99	22	95384	Cytoplasm	↓ 2.67829
TonB-dependent Receptor Plug domain protein	136	2	1310709	5.10	28	36470	Outer membrane	↓ 2.01854
Urocanate hydratase OS	353	3	470	5.63	26	59646	Cytoplasm	↓ 2.73441
60 kDa chaperonin, groL	193	4	903916	4.92	24	57189	Cytoplasm	↓ 1.98799
Serine hydroxymethyltransferase,	173	5	557600	5.44	31	45195	Cytoplasm	↓ 2.10096
3-oxoadipyl-CoA thiolase	270	6	1310697	5.89	51	41279	Cytoplasm	↑ 2.18868
Phenol hydroxylase P5 protein	124	7	1310911	4.53	18	39500	Cytoplasm	↑ Only in acetate + phenol
Pirin family protein OS	147	8	1310689	4.96	13	35227	Cytoplasm	↑ Only in acetate + phenol
Muconate and chloromuconate cycloisomerases family protein	196	9	1343073	5.41	27	39275	Cytoplasm	↑ Only in acetate + phenol
Catechol 1,2-dioxygenase, catA	140	10	1343073	4.70	37	33526	Cytoplasm	↑ 24.5042
MetA-pathway of phenol degradation family protein OS	81	11	1358412	5.30	26	32212	Outer membrane	↑ 9.57105
3-oxoadipate CoA-transferase subunit B (Fragment)	181	12	1310659	4.86	42	22789	Cytoplasm	↑ Only in acetate + phenol
3-oxoadipate CoA-transferase subunit A (Fragment)	149	13	1310720	7.66	46	19886	Cytoplasm	↑ Only in acetate + phenol
Uncharacterized protein	133	14	1148157	4.85	19	23098	Cytoplasm	↑ 1.81059
Nitroreductase family protein	118	15	1310689	5.83	41	21938	Cytoplasm	↑ Only in acetate + phenol
Fimbrial protein	205	16	575584	7.77	39	13897	Outer membrane	↑ Only in acetate + phenol
OsmC-like family protein	176	17	1310689	5.71	66	14449	Cytoplasm	↑ Only in acetate + phenol
Twitching mobility protein, pilT	40	18	1310605	6.27	23	35731	Cytoplasm	↑ Only in acetate + phenol
Cupin domain protein	112	19	1310689	5.37	25	26710	Cytoplasm	↑ Only in acetate + phenol
Isocitrate lyase	216	20	470	5.30	19	59646	Cytoplasm	↑ Only in acetate + phenol

^a^MOWSE scores of the highest confident matches (P < 0.05).

^b^The number refers to the spot numbers as given in Fig. 6.

^c^pI, the predicted isoelectric point calculated from the protein sequence.

^d^The predicted molecular weight calculated from the protein sequence.

In this study, 3-oxoadipyl-CoA thiolase was up-regulated by 2.18868-fold in bacteria grown on acetate plus phenol versus those grown on acetate alone. The function of 3-oxoadipyl-CoA thiolase is to catalyze the transformation of 3-oxoadipyl-CoA via the 3-oxoadipate pathway, forming acetyl-CoA. 3-oxoadipyl-CoA thiolases from various gram-negative have been biochemically characterized including those of *Pseudomonas knackmussii* B13^63^ and *P. putida* PRS2000^66^. To date, 3-oxoadipyl-CoA thiolases have been discovered in only a few gram-positive organisms, including *Rhodococcus opacus*1CP^67^ and *Rhodococcus jostii* RHA1^68^.

Interestingly, the enzyme of the MetA-pathway of phenol degradation family protein was up-regulated by 9.57105 fold in cells grown on phenol plus acetate compared with those grown on acetate alone. The specific function of this protein has not been documented.

Proteins from spot numbers 8, 15, 16, 17, 18, 19 and 20 (Table 3) were only expressed when bacteria were grown on acetate plus phenol, and these proteins included fimbrial protein. Previous studies have demonstrated that fimbrial proteins are generally insoluble and resistant to chemical and proteolytic degradation^69^, and this protein may be predicted to contribute to enhanced immune responses. Therefore, we can infer that high concentrations of phenol may be toxic to bacterial cells.

Other proteins not known to be involved in phenol catabolism were also upregulated in *Acinetobacter* sp. DW-1 grown on phenol-containing medium. These proteins included OsmC-like family proteins, which was consistent with the finding that the toluene catabolic plasmid pD2RT of *Pseudomonas migulae* strain D2RT, which encodes an OsmC-like protein is up-regulated when this bacterium is grown on toluene, Thus these proteins likely play a role in the detoxification of organic peroxides^70^.

Isocitrate lyase was also highly up-regulated in *Acinetobacter* sp. DW-1 grown on acetate supplemented with phenol. Isocitrate lyase has been reported to be responsible for the glyoxylate bypass, a unique metabolic pathway^71^, and it is also up-regulated during hexadecane degradation by *Acinetobacter oleivorans* DR1. These results suggest that bacterial cells need to synthesize cellular precursors from carbon at a rapid rate during detoxification of organic compounds^72^.

Nitroreductases, a group of enzymes that catalyze the reduction of nitro groups on aromatic compounds, were also upregulated in *Acinetobacter* sp. DW-1 grown on phenol-containing medium. These proteins have recently attracted enormous interest because of their central role in mediating nitroaromatic toxicity. In particular, they were mainly investigated in TNT^73^ and RDX^74^ degrading strains. However, the detailed mechanism of their involvement in degradation of phenol by bacteria has not been documented. Some studies have reported that nitroreductase family proteins could be induced in response to catechol, 2-methoxyhydroquinone, and chromanon^23, 28^, although other reports indicate that nitroreductase family proteins are not induced within a short time of phenol stress^28^. We hypothesize that the nitroreductase upregulation in *Acinectobacter* sp. DW-1 grown on acetate supplemented with phenol resulted from the decomposition of phenol into catechol, catalyzed by phenol hydroxylase.

Interestingly, the abundance of other proteins including Cupin domain protein OS and Twitching mobility protein were also increased in samples from bacteria grown on acetate plus phenol. To date, no correlation between these proteins and biodegradation of organic matter has been documented.

Five proteins were found to be downregulated in *Acinetobacter* sp. DW-1 grown on phenol-containing medium. Among these, aconitate hydratase, plays a critical role in the tricarboxylic acid (TCA) cycle by catalyzing the citrate to isocitrate reaction. The TCA cycle responsible for energy supply. The presence of phenol toxic to bacterial cells, leading to inhibition of their growth and metabolism and accounting for the reduction in this enzyme of the TCA cycle.

TonB-dependent receptors have been reported to mediate the transport of siderophore into the periplasm of gram-negative bacteria. A complex of three membrane-spanning proteins TonB, ExbB, and ExbD couples the chemiosmotic protential of the cytoplasmic membrane with siderophore uptake across the outer membrane. The function of 60 kDa chaperonin is to prevent misfolding and promote refolding and proper assembly of unfolded polypeptides generated under stress conditions (http://www.uniprot.org/uniprot/A0A010LHT0). Elucidation of the reason for its downregulation as a result of exposure to phenol will require further study.

### Transcriptome sequences assembly and analysis

To reveal the mechanism of phenol biodegradation by *Acinetobacter* sp. DW-1, we performed comparisons of the transcriptome of *Acinetobacter* sp. DW-1 grown on acetate and acetate plus phenol. The high-throughput RNA-seq generated a total length of 1208514000 and 1792215250 clean reads from control samples CK-1and CK-2, respectively. A total length of 1459341500 and 1393273750 clean reads was obtained from samples T-1 and T-2, respectively. The Q20 of all of these samples reached 97%, which indicated the high quality of the transcriptome sequencing. Biosample (SAMN05919934, SAMN05919933, SAMN05792302 and SAMN05710740) and Bioproject (PRJNA350871) information has been submitted to NCBI.

**Figure 7:**
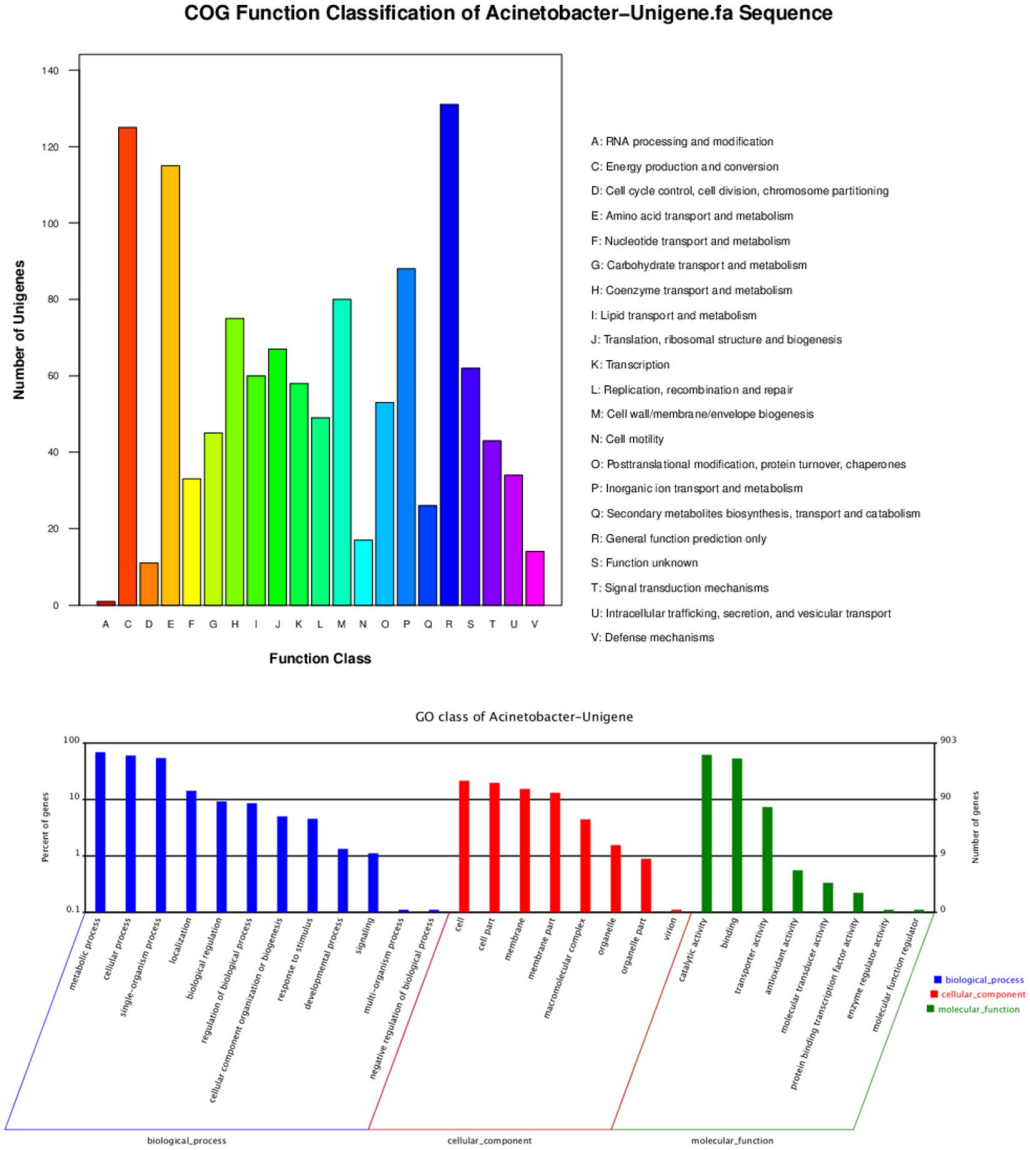
GO and COG function classifications of the unigenes of CK and T samples.

Subsequently, 959 unigenes were annotated and divided into 21 cluster of orthologous groups of proteins (COG) (Fig. 7). The largest cluster was R (general function prediction only), followed by C (energy production and conversion). These unigenes are known to be involved in the degradation and metabolism of benzene rings, the TCA cycle, amino acids and lipids, and they are involved in the metabolic processes of *Acinetobacter* sp. DW-1. The relatively high frequency of S (function unknown) indicates that many novel genes still require further identification in *Acinetobacter* sp. DW-1. The GO database annotated 903 unigenes (Supplementary Table S1). These unigenes were classified as having biological process, cellular component, and molecular functions. The high proportion of unigenes involved in catalytic activity function, binding, metabolic processes, cellular processes and single-organism processes implied the presence of various types of secondary metabolites and a complex regulation mechanism in phenol biodegradation by *Acinetobacter* sp. DW-1(Supplementary Table S2).

Differentially expressed genes (DEGs) were observed during phenol biodegradation. Among the DEGs, 694 genes were found to be differentially up-regulated genes (DUGs), and 340 genes were identified as differentially down-regulated genes (DDGs). The data, including the gene ID, gene length, FRKM in the control and treatment groups, log2FC (T/CK), FDR, and annotation of all of the DEGs are provided in Supplementary Table S3. GO analysis indicated that DEGs were members of 22 classes. The number of up-regulated genes in each functional category is shown in Supplementary Table S4. Among the 694 up-regulated genes, genes encoding metabolic processes comprised the highest percentage (201 genes, approximately 16% of all up-regulated genes), whereas genes related to catalytic activity comprised approximately 15% of all up-regulated genes (182 genes). Genes encoding proteins involved in cellular processes (179 genes), binding (155 genes), single-organism processes (146 genes), cell (80 genes), cell parts (75 genes), and membrane (39 genes) were also up-regulated. In addition, genes classified in the category of genes of unknown function represented a considerable portion of the induced genes (26 genes). The numbers of down-regulated genes in each functional category are shown in Supplementary Table S4. Among the down-regulated genes, those involved in metabolic processes were the dominant category (63 genes; 15%), followed by genes involved in single-organism processes (56 genes; 10%), catalytic activity (51 genes), binding (48 genes), and cellular processes (45 genes), and membrane (22 genes).

Additionally, 331 DEGS were annotated by the KEGG database. The high proportion of metabolic pathways (19.94%) and the biosynthesis of secondary metabolite pathways (10.88%) among all of the DEGs indicated the production of various types of metabolites during phenol biodegradation process (Supplementary Table S5). It is more interesting that phenol degradation pathway only belongs to metabolic pathways. During phenol biodegradation, genes involved in responses to stimuli showed significantly increased expression, including Unigene0000224, Unigene0000852, Unigene0002231, Unigene0001700, Unigene0000948, Unigene0000663 and Unigene0001896. Thus high concentrations of phenol cause physiologic stress in cells of *Acinetobacter* sp. DW-1.

The phenol degradation pathway is a part of the benzoate degradation pathway in the KEGG database (ko00362), and crucial genes in this metabolic pathway were found to be up-regulated (Table 4), including Unigene0000836 (phenol hydroxylase), Unigene0001131 (catechol 1, 2-dioxygenase), Unigene0001341 (muconate cycloisomerase), Unigene0000980 (3-carboxy-cis,cis-muconate cycloisomerase) and Unigene0000678(3-oxoadipate CoA-transferase). The MetA-pathway of phenol degradation family protein was also up-regulated by 2.3-fold compared to the expression in the CK groups. In addition, phenol degradation protein involved in meta-pathway of phenol degradation was up-regulated 37.3-fold. It has been reported that cells grown on sufficiently high concentrations of sodium benzoate simultaneously induce catechol 1,2-dioxygenase and catechol 2,3-dioxygenase, indicating that they can degrade sodium benzoate through both the ortho- and the meta- cleavage pathways^75^. In addition, proteome analysis indicates that *Pseudomonas* sp. K82 has three main metabolic pathways for degradation of aromatic compounds, and could induce a specific metabolic pathway for each compound. Moreover, the catechol 2, 3-dioxygenase pathway was the major pathway induced by aniline exposure in *Pseudomonas* sp. K82 and the catechol 1, 2-dioxygenase pathway was the secondary pathway whereas the catechol 1,2-dioxygenase pathway was the major pathway induced by benzoate exposure^76^. In addition, the results of enzyme assays showed that the efficiency of biodegradation of phenol by meta-pathways was higher than that by ortho-pathways^77^. Therefore, we infer from the data produced in the present study that *Acinetobacter* sp. DW-1 utilizes phenol primarily through the orth-pathway, but that it can also degrade phenol through the meta-pathway in some conditions.

**Table 4:** Functions of key genes detected by RNA-Seq.

Gene ID	Description	COG no.	Fold changes (log2 Ratio(T/CK))	KEGG no.	EC no.	Predicted function
**Energy production and conversion**						
Unigene0002417	phenol degradation protein	COG4313	5.217441542	N/A	N/A	Protein involved in meta-pathway of phenol degradation
Unigene0002240	bacterial Cytochrome Ubiquinol Oxidase family protein	COG1271	4.535288588	K00425	[EC:1.10.3.-]	establishment of localization
Unigene0001576	phenol 2-monooxygenase	COG2871	6.942107699	N/A	N/A	oxidoreductase activity
Unigene0002249	ATP F0F1 synthase subunit alpha	COG0056	2.197069232	K02111	[EC:3.6.3.14]	cation-transporting ATPase activity
**Secondary metabolites biosynthesis, transport and catabolism**						
Unigene0001131	catechol 1,2-dioxygenase	COG3485	4.94	K03381	[EC:1.13.11.1]	catechol-containing compound metabolic process
Unigene0000378	protocatechuate 3,4-dioxygenase	COG3485	5.587102309	K00448	[EC:1.13.11.3]	cellular aromatic compound metabolic process
Unigene0001606	coenzyme F390 synthetase	COG1541	−15.2013869	K01912	[EC:6.2.1.30]	aromatic compound catabolic process
Unigene0000908	3-carboxy-cis,cis-muconate cycloisomerase	COG0015	4.01609738	N/A	N/A	catechol-containing compound catabolic process
Unigene0000803	benzoate 12 dioxygenase alpha subunit	COG4638	−2.879859554	K05549	[EC:1.14.12.10]	cellular aromatic compound metabolic process
Unigene0001123	ligand-gated channel protein	COG4774	−3.646712089	K02014	N/A	iron chelate transport
Unigene0000980	acetyl-CoA acetyltransferase	COG0183	−1.173807482	K00632	[EC:2.3.1.16]	Acetyl-CoA acetyltransferase
Unigene0000678	3-oxoadipate CoA-transferase	COG1788	4.294322399	K01031	[EC:2.8.3.6]	Acyl CoA:acetate/3-ketoacid CoA transferase, alpha subunit
Unigene0001528	ABC transporter ATP-binding protein, partial	COG1127	1.998799132	K02065	N/A	ABC-type transport system involved in resistance to organic solvents
**Cell wall/membrane/envelope biogenesis; General function prediction only**						
Unigene0001341	muconate cycloisomerase	COG4948	2.49	K01856	[EC:5.5.1.1]	L-alanine-DL-glutamate epimerase and related enzymes of enolase superfamily
Unigene0000039	nitroreductase family protein	COG3560	4.047415045	K07078	N/A	catalytic activity
Unigene0000969	quercetin 2,3-dioxygenase	COG1741	3.101493853	K06911	N/A	None predicted
**Posttranslational modification, protein turnover, chaperones**						
Unigene0000948	osmotically inducible protein C	COG1765	3.54518103	K07397	N/A	response to stress
**Translation, ribosomal structure and biogenesis; Inorganic ion transport and metabolism**						
Unigene0002513	50 S ribosomal protein L10, partial	COG0244	2.634136949	K02864	N/A	rRNA binding; structural molecule activity
Unigene0001303	50 S ribosomal protein L2	COG0090	2.264955891	K02886	N/A	RNA binding; structural molecule activity
Unigene0000305	30 S ribosomal protein S13	COG0099	2.001593462	K02952	N/A	RNA binding; structural molecule activity
Unigene0000065	30 S ribosomal protein S3, partial	COG0092	2.392017692	K02982	N/A	poly(A) RNA binding; structural molecule activity
Unigene0000602	50 S ribosomal protein L7/L12	COG0222	2.765846319	K02935	N/A	None predicted
Unigene0000077	ATPase, partial	COG2217	5.545384277	K01533	[EC:3.6.3.4]	transition metal ion binding,copper-transporting ATPase activity
Unigene0000176	E1-E2 ATPase	COG2217	3.350705804	K01533	[EC:3.6.3.4]	intrinsic component of membrane, copper ion transmembrane transport
**Coenzyme transport and metabolism; Lipid transport and metabolism**						
Unigene0001464	lipoyl synthase	COG0320	−3.178920684	K03644	[EC:2.8.1.8]	cation binding; sulfurtransferase activity;
Unigene0001096	3-oxoacyl-ACP synthase	COG0332	1.099154317	K00648	[EC:2.3.1.180]	fatty acid metabolic process
Unigene0002287	fatty acid desaturase family protein	COG3239	1.092701954	K00508	[EC:1.14.19.3]	none predicted
**Transcription; Defense mechanism; Signal transduction mechanisms**						
Unigene0001039	LysR family transcriptional regulator	COG0583	2.992016687	N/A	N/A	cellular macromolecule biosynthetic process
Unigene0000056	TetR family transcriptional regulator	COG1309	−0.379311349	NA	NA	nucleic acid binding
Unigene0000663	Predicted transcriptional regulator,BolA superfamily	COG5007	3.489769782	NA	NA	cellular response to stress
Unigene0000886	AraC family transcriptional regulator	COG2207	−1.360719141	NA	NA	DNA binding
Unigene0001910	GntR family transcriptional regulator	COG2186	−0.97172089	K05799	NA	transcriptional repressor for pyruvate dehydrogenase complex
Unigene0002400	CRP/FNR family transcriptional regulator	COG0664	−0.045371841	K10914	NA	nucleic acid binding
Unigene0000781	HTH-type transcriptional regulator	COG0789	1.939857402	N/A	N/A	regulation of transcription, DNA-templated
Unigene0000852	serine hydrolase	COG1680	1.512798695	N/A	N/A	response to toxic substance
**No related COG**						
Unigene0000054	phenol hydroxylase	COG0543	15.87929606	K03380	[EC:1.14.13.17]	2-polyprenylphenol hydroxylase and related flavodoxin oxidoreductases
Unigene0002446	metA-pathway of phenol degradation family protein	N/A	1.177456264	N/A	N/A	None predicted
Unigene0001290	biofilm synthesis protein	N/A	4.094059655	N/A	N/A	transmembrane transporter activity
Unigene0001394	fimbrial protein	N/A	15.21820373	N/A	N/A	cell projection
Unigene0000307	membrane protein	N/A	4.679820328	N/A	N/A	intrinsic component of membrane
Unigene0002168	Fis family transcriptional regulator	COG1690	−6.6691460517	K14415	NA	gene expression
Unigene0000243	LuxR family transcriptional regulator	NA	−0.652472163	NA	NA	nucleic acid binding

N/A: Not available.

Protocatechuate 3,4-dioxygenase, which catalyzes the aromatic ring cleavage of 3,4-dihydroxy benzoate by incorporating both atoms of molecular oxygen to yield β-carboxy-cis,cis-muconate was highly up-regulated. Nitroreductase is typically reported to be up-regulated in the presence of toxic substances. Biofilm synthesis protein and membrane protein were strongly up-regulated. Biofilm synthesis proteins such as Unigene0001290 and Unigene0001946 were up-regulated. Biofilm synthesis protein is predicted to have transmenbrane transporter activity and thus to be responsible for nutrient transport and processing. In addition, many membrane proteins were also found markedly up-regulated. For instance, the membrane proteins which were responsible for protein binding (Unigene0000178), establishment of localization (Unigene0000385), transition metal ion binding (Unigene0000415), nucleic acid binding(Unigene0001807), and iron-sulfur cluster binding (iron-sulfur cluster binding). Furthermore, integral membrane TerC family protein (Unigene0002265), outer membrane autotransporter barrel domain protein (Unigene0000415) and outer membrane OmpA family protein (Unigene0001866) were also up-regulated. Additionally, it is reported that some cell surface proteins such as fimbrial protein were found to be implicated in biofilm formation^78^. In this study, fimbrial protein was markedly up-regulated in both 2D-gel based proteomic analysis and RNA-seq analysis. In particular, its expression was 38,165-fold higher than that the CK groups in transcriptome analysis. It is reported that fimbrial protein could confer the ability to bind to specific surfaces^79^, and thus its upregulation makes biofilm easier to form. To the best of our knowledge, the up-regulation of membrane proteins were to defense the toxicity from higher concentration of phenol^22^. High concentrations of phenol as a toxicant might stimulate immune responses and the defense function of cells, which leads to the formation of biofilm. Additionally, there were many transcriptionl regulators were involved in phenol biodegradation (Table 4), such as LysR/AraC/LuxR/TetR/Fur/GntR/Fis/CRP/FNR/CysB family transcriptional regulators, even some putative transcriptional regulator. LysR family transcriptional regulators were reported to be activators of expression of genes encoding enzymes that are associated with ortho-cleavage through the β-ketoadipate pathway, including the gene of *catBCA* ^80, 81^. In this study, LysR family transcriptional regulators was detected up-regulate similarly. Preliminary data for AraC family transcriptional regulators indicated that they were involved in expression control of a catabolic operon, and generally acted as transcription activators in the presence of a chemical effector molecule^82^. There was few example about TetR family involved in aromatic compouds^83^. In this study, TetR family transcriptional regulator was down-regulate, the result was in accord with the primary data TetR-type regulator was a repressor for the expression of the genes for aromatic compounds^84–86^. GntR family members were also assumed as repressors to control the degradation of aromatic compounds in the absence of the pathway substrate^81^. In our study, GntR family transcriptional regulator was detected down-regulate 1.96 fold, which was consistent with previous reports that it played a repressor. There was little information about the CRP/FNR family transcriptional regulator involved in aromatic compounds. It has been reported that the CRP/FNR regulator activates expression of the hbaR gene which allowed transcription of the 4-hydroxybenzoate degradation genes in *Rhodopseudomonas palustris* under anaerobic conditions^87^. However, in our study, the function of the FNR regulator need to be further verified. Fis regulators are global regulators of transcription in *Escherichia coli* in the adjustment of cellular metabolism to varying growth. But the function of Fis regulators during phenol biodegradation need to be further studied^88^. Similarly, according to preliminary data, the LuxR-type regulators are generally functioning as transcriptional activators, the accurate function of this regulator in phenol metabolism will attract more attention^89^. HTH-type transcriptional regulator always bind transcriptional activators with the target DNA, which induce transcription initiation by interacting with the aromatic substrate^90^. In addition, the genes related basic metabolism were also increased, including 50 S ribosomal protein, 30 S ribosomal protein, ATPase and fatty acid desaturase family protein. However, some stress response genes were up-regulated, which indicated the toxicity of phenol to cells during phenol degradation process.

### Validation of RT-qPCR

**Figure 8:**
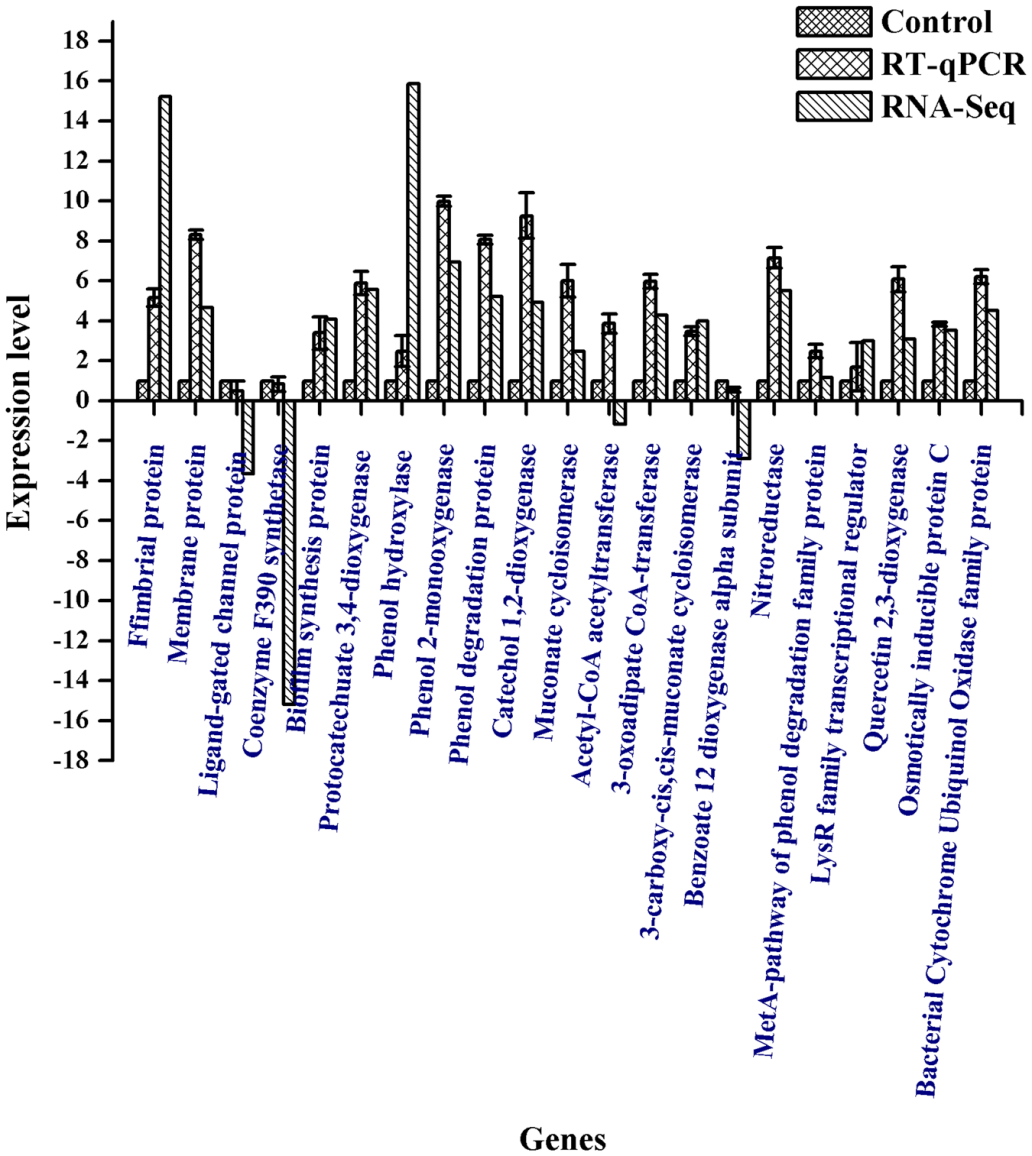
Validation of transcriptome data by quantitative RT-PCR analysis. Log2 fold changes calculated with both RT-qPCR and RNA-Seq analyses

The RNA-seq data were validated by RT-qPCR selecting 22 DEGs (Table 4) that were crucial for phenol biodegradation. The log2 fold change in gene expression between the two conditions demonstrated significant correlation between RNA-seq and RT-qPCR. In particular, the results for the relative expression of these genes using RNA-seq and RT-qPCR showed over 80% consistency, indicating the reliability of the RNA-seq analysis (Fig. 8).

### Metabolism pathway analysis

**Figure 9:**
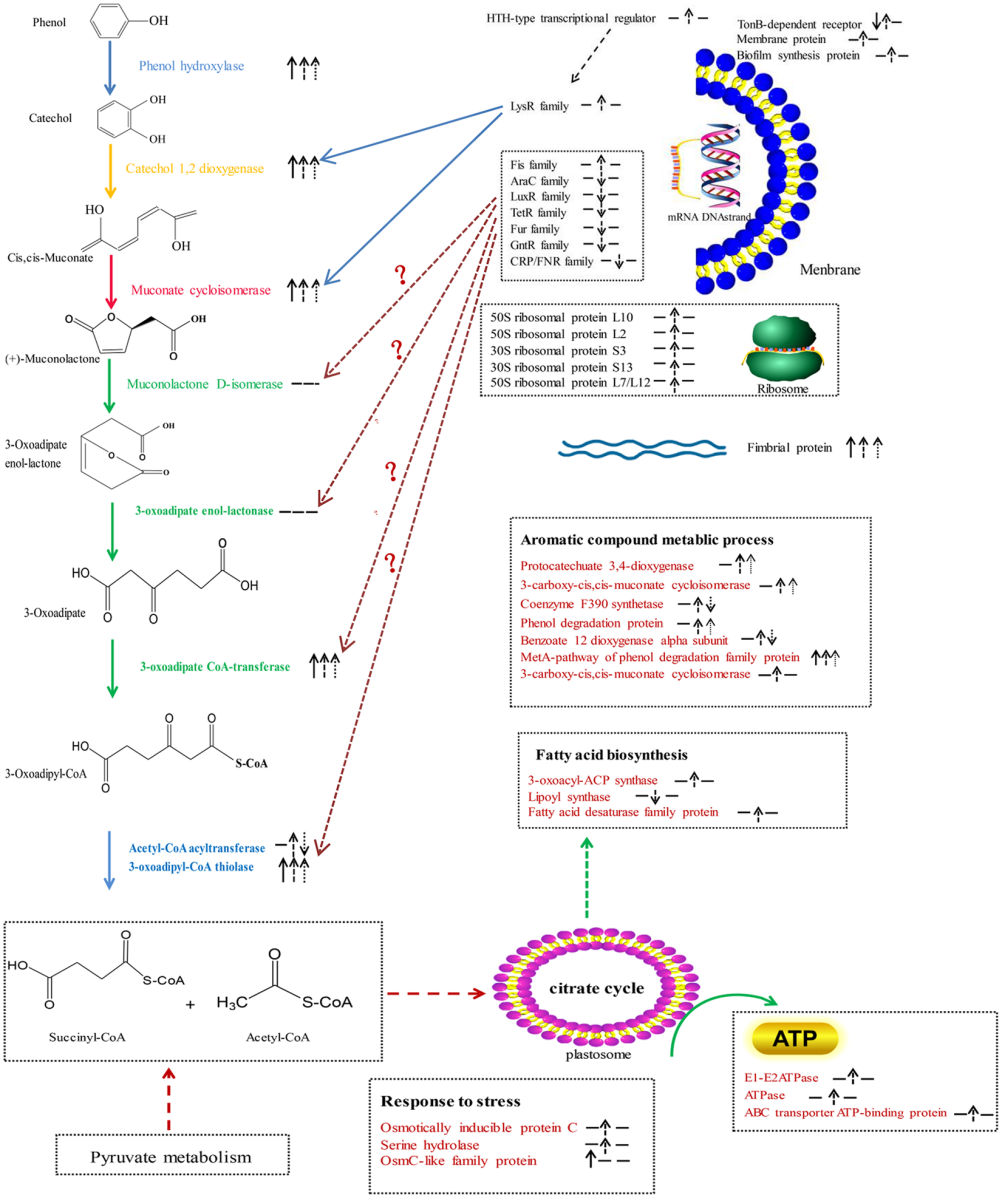
The metabolic pathway of phenol biodegradation. The proteins and genes identified by 2-D electrophoresis and RNA-seq, and the variation of them in the state of regulation are indicated by arrows(up/down), “”represents the state of proteins detected by 2-D electrophoresis, “”represents the state of genes detected by RNA-seq. “”represents the state of proteins or genes validated by RT-qPCR. “−” represents none detected.

According to the identified catabolic proteins and genes, phenol metabolism related genes were constructed basing on the location of the protein after searching in the KEGG database as shown in Fig. 9. The most important phenol catabolic enzymes were identified: phenol hydroxylase, catechol 1, 2-dioxygenase, muconate cycloisomerases, 3-oxoadipate CoA-transferase, acetyl-CoA acyltransferase, 3-oxoadipyl-CoA thiolase. In addition to these genes, transcriptional regulator indicated obvious expression in Table 4 and Fig. 9, which involved in regulating the expression of key genes in phenol metabolic pathway, and the function of some new transicriptional regulators need to be further confirmed. Meanwhile, membrane related proteins such as biofilm synthesis protein and membrane protein showed increased expression, ribosomal proteins also upregulated differentially. Results of pathway analysis indicated that this strain catabolized phenol via the β-ketoadipate pathway, also known as the “ortho” route. This is consistent with previous studies, indicating that *Acinetobacter* sp. usually catalyze phenol as sole carbon and energy source by the ortho-cleavage pathway^13, 91^. However, some genes related aromatic compound metabolic process were found up-regulated, especially the phenol degradation protein which involved in the meta-pathway of phenol degradation. Thus, we could infer that meta-cleavage pathway may exist.

In summary, *Acinetobacter* sp. DW-1 has the potential to be applied in bioremediation. From the proteomic and transcriptomic analysis, we determined that *Acinetobacter* sp. DW-1 degraded phenol mainly by the ortho pathway because of the induction of phenol hydroxylase and catechol-1, 2-dioxygenase. There is also evidence for induction of the meta-pathway of phenol biodegradation by *Acinetobacter* sp. DW-1, as the expression of phenol degradation protein involved in meta-pathway of phenol degradation increased. Despite the abundance of catabolic proteins, some novel candidate proteins (OsmC-like family protein, MetA-pathway of phenol degradation family protein, fimbrial protein, coenzyme F390 synthetase, nitroreductase family protein) and low abundance transcriptional regulators (GntR/LuxR/CRP/FNR/TetR/Fis family transcriptional regulator) were successfully identified, especially MetA-pathway of phenol degradation family protein, it is firstly reported dramatically up-regulated in phenol biodegradation, and the specific function of this protein need to be further studied. In addition, fimbrial protein in outer membrane was also dramatically up-regulated and has not been reported in phenol degradation related literatures. FNR and Fis family transcriptional regulators were also not documented before during phenol biodegradation. These findings provide novel insights and improved understanding of the phenol biodegradation mechanisms of *Acinetobacter* sp. DW-1. However, further study of the detailed roles and factors regulating these proteins in *Acinetobacter* sp. and of mechanism of phenol biodegradation are urgently required.

## Conclusion

This study provides a detailed view of biofilm formation of *Acinetobacter* sp. DW-1 on a carrier material consisting of polyhedron hollow polypropylene balls. The biofilm activity was optimal when the cells were immobilized for approximately 72 h. The phenol degradation test indicated that the immobilized *Acinetobacter* sp. DW-1 cells exhibited high phenol degradation ability and thus could potentially be applied to bioremediation in phenol-polluted drinking water. Additionally, it is the first time to simultaneously use both proteomic and transcriptomic approaches to investigate phenol metabolism. The transcriptomics and proteomics analysis data indicated that there was a significant correlation between them. However, transcriptomic analysis obtained less-soluble membrane proteins and low-abundance transcriptional regulators which had not been found in proteomic analysis with its inherent limitation. Whereas, proteomic analysis verified the expression of genes which were also detected by transcriptome sequencing. Omics analysis showed that *Acinetobacter* sp. DW-1 degrades phenol mainly by the ortho pathway, some novel candidate proteins (OsmC-like family protein, MetA-pathway of phenol degradation family protein, fimbrial protein and coenzyme F390 synthetase) and transcriptional regulators (GntR/LuxR/CRP/FNR/TetR/Fis family transcriptional regulator) were identified to be potentially involved in phenol biodegradation, especially MetA-pathway of phenol degradation family protein and fimbrial protein showed a strong positive correlation with phenol biodegradation, and Fis family transcriptional regulator is likely to exert its effect as activators of gene expression. However, the specific function of these candidate proteins and genes in phenol biodegradation requires further confirmation.

## Electronic supplementary material

Supplementary Information

Supplementary Table S1

Supplementary Table S2

Supplementary Table S3

Supplementary Table S4

Supplementary Table S5
