# Supplementary material for: Acinetobacter sp. DW-1 immobilized on polyhedron hollow polypropylene balls and analysis of transcriptome and proteome of the bacterium during phenol biodegradation process
Source: Sci Rep. 2017 Jul 7;7:4863. doi: 10.1038/s41598-017-04187-6 (PMC5501837; doi:10.1038/s41598-017-04187-6)
Supplement: Supplementary file 1 — Supplementary Information [file 41598_2017_4187_MOESM1_ESM.pdf]

# *Supplementary Information*

# *Acinetobacter* sp.DW-1 immobilized on polyhedron hollow polypropylene balls and analysis of transcriptome and proteome of the bacterium during phenol biodegradation process

Qihui Gu[1, 2], Qingping Wu[2]*, Jumei Zhang[2], Weipeng Guo[2], Huiqing Wu[2], Ming Sun[2]

*** Correspondence:** Qingping Wu: E-mail: wuqp203@163.com

## 1. Supplementary methods

### 1.1 Antimicrobial susceptibility test

19 antibiotics: kanamycin(K; 30 μg), streptomycin(S; 10 μg), tetracycline(TE; 30 μg), ampicillin(AMP; 10 μg), chloramphenicol (C; 30 μg), ciprofloxacin(CIP; 5 μg), meropenem(MEM; 10 μg), cephalothin(KF; 30 μg), tobramycin(TOB; 10 μg), gentamicin(CN; 10 μg), ceftazidime(CAZ; 30 μg), cefotaxime(CTX; 30 μg), imipenem(IPM; 10 μg), cefepime(FEP; 30 μg), trimethoprim-sulfamethoxazoie (SXT; 23.75/1.25 μg), ampiclllin(AMP; 10 μg), levofloxacin(LEV; 5 μg), aztreonam(ATM; 30 μg).

### 1.2 Phenol degradation by free cells and immobilized cells

*Acinetobacter* sp. DW-1 cells were inoculated into R2A fluid nutrient medium for 24 h; then, 100 μL of R2A-preculture was respectively inoculated into two Erlenmayer flasks (500 mL) containing MSM supplemented with phenol (4 mM) as the sole carbon source. Among of them, one Erlenmayer flask with polyhedron hollow polypropylene balls, the other without polyhedron hollow polypropylene balls. After static cultivation culture for 96 h, 1 mL of medium was withdrawed from Erlenmayer flask which without polyhedron hollow polypropylene balls, the biomass concentration of free cells was determined with a Bradford Protein Assay Kit (Sangon, Shanghai, China). In the other Erlenmayer flask, biofilm obtained from polyhedron hollow polypropylene balls after ultrasonication was diluted with PBS buffer at an appropriate. Similarly, the biomass concentration of the biofilm was also detected as above. Equal amounts of free cells and immobilized cells were inoculated to the bottles containing MSM supplemented with phenol (4 mM), and then performed to degradation phenol.

## 2. Supplementary Figures

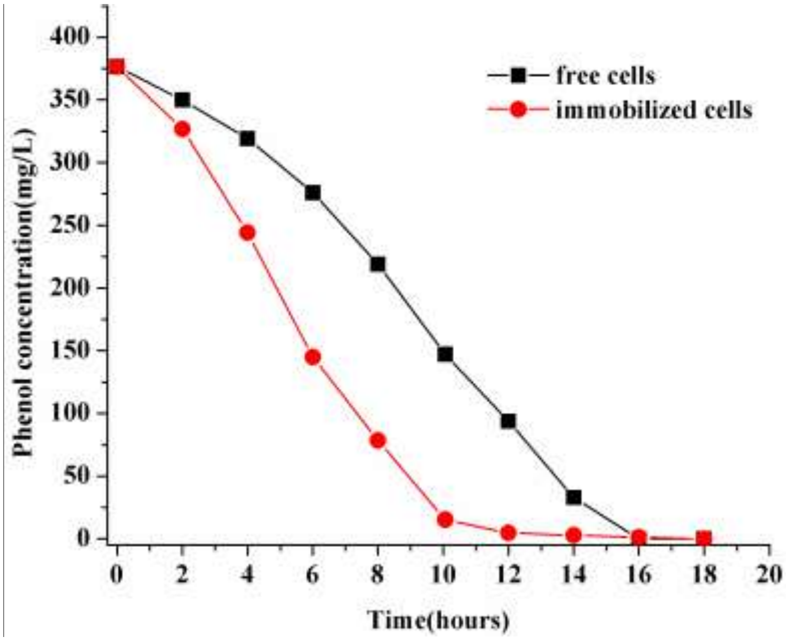


Fig.S1 Phenol degradation by free cells and immobilized cells of *Acinetobacter* sp. DW-1

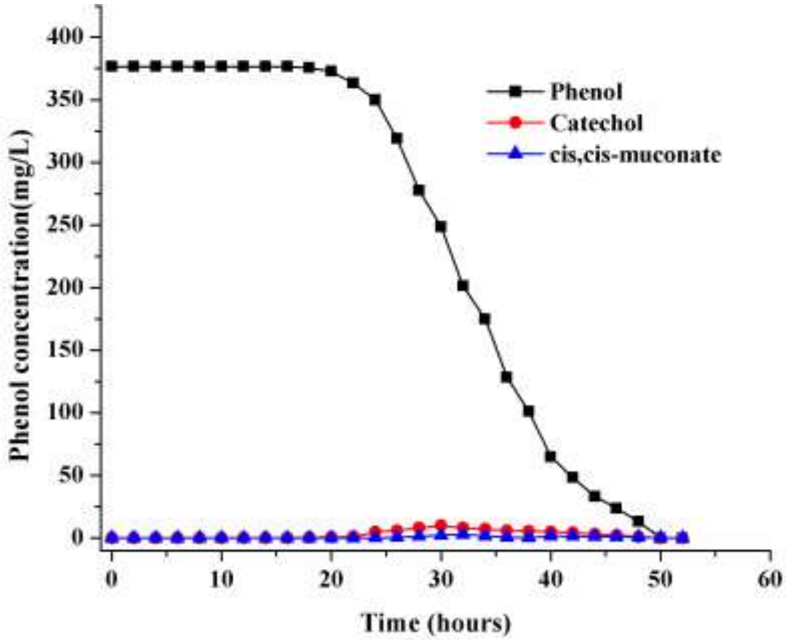


Fig.S2 Detection of phenol, catechol and cis,cis-muconate during biodegradation by *Acinetobacter* sp. DW-1

## 3. Supplementary Tables

Table S6 Primers for RT-qPCR

| Seq ID | Orientation | tm | GC% | Seq length | Seq |
|---|---|---|---|---|---|
| Unigene0000836 | FORWARD | 59.31 | 50 | 248 | CAGCCAGAAGAACTTGCTCA |
| Unigene0000836 | REVERSE | 60.38 | 50 | | ATCGCCTTCATAAGCAGCAC |
| Unigene0002417 | FORWARD | 60.04 | 45 | 409 | GCTGGCATCAAGGAAATCAT |
| Unigene0002417 | REVERSE | 59.14 | 45 | | TTGGCTTCAACTGGATGTGT |
| Unigene0002446 | FORWARD | 60.05 | 40 | 249 | TTGCCACTGGAAATGATGAA |
| Unigene0002446 | REVERSE | 59.57 | 45 | | AGCGCATGGTAATTGATGTG |
| Unigene0000378 | FORWARD | 59.69 | 45 | 258 | GTGTTTGACGGATTGGGTTT |
| Unigene0000378 | REVERSE | 60.01 | 45 | | TTTTTGCTCACGCGTATCTG |
| Unigene0001253 | FORWARD | 60.57 | 50 | 288 | AACTTGGACTGCGCTTTCAG |
| Unigene0001253 | REVERSE | 60.24 | 45 | | TCCAAGTTGCAGGAACATCA |
| Unigene0001290 | FORWARD | 59.63 | 50 | 244 | CAGCGAGTTTTCTCAACGTG |
| Unigene0001290 | REVERSE | 60.78 | 50 | | ACACAGCATCAAGCTCAGCA |
| Unigene0000908 | FORWARD | 58.33 | 50 | 319 | GGCAGAAGCTGTAATGATGG |
| Unigene0000908 | REVERSE | 60.06 | 50 | | TGCAGGTTTTGTGCTACTGC |
| Unigene0000803 | FORWARD | 60.03 | 50 | 414 | TTTCTGCTGTGCACTGGAAC |
| Unigene0000803 | REVERSE | 59.97 | 50 | | TTTACCCCAAGAACCTGCAC |
| Unigene0002240 | FORWARD | 60.02 | 50 | 232 | TGCTCCTGCTCCGTTTACTT |
| Unigene0002240 | REVERSE | 59.74 | 45 | | TTGGTTGTTGAACGAGTTGC |
| Unigene0000948 | FORWARD | 59.53 | 50 | 252 | GAAATCACCAGTGGTCGTCA |
| Unigene0000948 | REVERSE | 60.02 | 55 | | CGGCTAAACTTCCAGTGAGC |
| Unigene0000969 | FORWARD | 58.41 | 45 | 332 | CAGGTCACGGAATTTCACAT |
| Unigene0000969 | REVERSE | 60.57 | 50 | | CATGTTGCGCTTAGCATCTG |
| Unigene0001039 | FORWARD | 60.08 | 45 | 399 | TTGCATGATGACATCGACCT |
| Unigene0001039 | REVERSE | 59.84 | 42.86 | | CACCAGACAATGATCGGTTTT |
| Unigene0001606 | FORWARD | 60.43 | 55 | 239 | GAGCTTCGAGAGCTGCAAAC |
| Unigene0001606 | REVERSE | 59.8 | 45.45 | | TGTACACCTGCTTCATCGAACT |
| Unigene0001123 | FORWARD | 59.95 | 50 | 230 | GCGGCGTTCCATATACAGAT |
| Unigene0001123 | REVERSE | 60.18 | 50 | | TCACGAGTTTGAGCTGTTGC |
| Unigene0001576 | FORWARD | 59.94 | 50 | 254 | GGGTGGTTCAGGTCTTTCAA |
| Unigene0001576 | REVERSE | 59.34 | 55 | | TAGCTCTGCCAAGTCTCGTG |
| Unigene0001131 | FORWARD | 60.1 | 45 | 256 | GCAGATGAAGCTGATGCAAA |
| Unigene0001131 | REVERSE | 59.32 | 45 | | CGCAAAACCAACAGATTCAG |
| Unigene0001341 | FORWARD | 59.93 | 55 | 310 | TCCAACTATCCGTCCTCACC |
| Unigene0001341 | REVERSE | 60.61 | 50 | | TTCACCCCAACCGACAATAC |
| Unigene0000980 | FORWARD | 60.1 | 50 | 257 | ACCATTGCTCCGATTAGTGC |
| Unigene0000980 | REVERSE | 59.57 | 40 | | ACCATTGCAAAAGCCTCATT |
| Unigene0000678 | FORWARD | 59.68 | 50 | 380 | GACCTGACCATTGTGAGCAA |

| | | | | | |
|---|---|---|---|---|---|
| Unigene0000678 | REVERSE | 59.39 | 50 | | TCAGACTGACGTGGGAAAGA |
| Unigene0001394 | FORWARD | 60.14 | 50 | 244 | CTGAGGCTGTCTGCAAATGA |
| Unigene0001394 | REVERSE | 60.69 | 55 | | GCCCGAACTAGTTGAACACG |
| Unigene0000307 | FORWARD | 59.84 | 45 | 239 | GGCAAGTGAAATTTGGTGGT |
| Unigene0000307 | REVERSE | 59.81 | 45 | | TTCTGCCGAAAATGGAGACT |
